# Alkali-Activated Mortars Reinforced with *Arundo donax*: Properties and Durability to Environmental Stresses

**DOI:** 10.3390/ma16113898

**Published:** 2023-05-23

**Authors:** Stefania Manzi, Luisa Molari, Grazia Totaro, Andrea Saccani

**Affiliations:** Department of Civil, Chemical, Environmental, and Materials Engineering, University of Bologna, Via Terracini 28, 40131 Bologna, Italy; luisa.molari@unibo.it (L.M.); grazia.totaro@unibo.it (G.T.); andrea.saccani@unibo.it (A.S.)

**Keywords:** alkali-activated binders, natural fibers, composites, durability

## Abstract

Natural fibers were used to modify alkali-activated fly-ash mortars. *Arundo donax* is a common, fast-growing, widespread plant with interesting mechanical properties. Short fibers of different lengths (from 5 to 15 mm) were added at a 3 wt% ratio to the binder amount to the alkali-activated fly-ash matrix. The possible effects on the fresh and cured properties of the mortars deriving from the different lengths of the reinforcing phase were investigated. The flexural strength of the mortars increased by up to 30% at the longest fiber dimensions, while the compressive strength remained almost unchanged in all of the compositions. The dimensional stability was increased slightly upon the addition of the fibers, depending on the fiber length, while the porosity of the mortars was reduced. Moreover, contrary to what was expected, the water permeability was not increased by the fibers’ addition, irrespective of their length. The durability of the obtained mortars was tested through freeze–thaw and thermo-hygrometric cycles. The results obtained so far underline a fair resistance to the changes in temperature and moisture and a better resistance to the freeze–thaw stresses of the reinforced mortars.

## 1. Introduction

The urge to replace materials with a high carbon dioxide footprint drives the search for alternative binders as substitutes for the long-established Portland cement. The use of alkali-activated binders is a promising approach, since they can be derived by activating industrial wastes of different origins. Among many possible sources, ground granulated blast-furnace slag (GGBFS) [1], discarded glasses unfit to be used in the production of new glass items [2], fly ash [3,4], and red mud [5] have been used to formulate alkali-activated binders. However, these materials suffer from extreme brittleness and low dimensional stability, as reported in the literature [6,7,8]. The most frequently applied remedy to these drawbacks is the addition of fibers. Although synthetic organic and inorganic fibers such as glass [9], carbon [10,11], recycled carbon [12], and poly(vinyl alcohol) (PVA) [13,14] can be used, a further environmental benefit can be obtained by using natural renewable fibers. Natural fibers have lower density and hardness, and some of them have comparable mechanical properties in terms of elastic modulus and tensile strength to those of the synthetic ones. Kenaf, sisal, cotton, flax, banana, and bamboo fibers are some of the most promising types that have been used to formulate reinforced alkali-activated materials, but many others have been employed [15,16,17,18,19,20,21].

However, the use of natural fibers has some drawbacks. The external surface can contain waxes, pectin, and other impurities that can compromise the adhesion of the fibers to the binding matrix. Moreover, the presence of hemicellulose does not contribute to the mechanical properties of the fibers, but instead increases their hydrophilicity, a feature that could be detrimental to the durability of the formulated composite. Therefore, before mixing, the fibers are usually treated in an alkaline solution in order to improve the final properties of the composite [22,23,24]. However, the evaluation of natural-fiber-reinforced composites’ durability has been seldom investigated [25,26,27,28] and remains incompletely understood.

In the present research, *Arundo donax* fibers were used to modify an alkali-activated matrix based on fly ash. *Arundo donax* has remarkable mechanical properties that have been investigated previously [29,30] and that make this material quite interesting in the building industry. This plant has been used in the past to formulate organic polymer-based composites [31,32], or even to produce hybrid composites with an inorganic/organic matrix [33]. The *Arundo donax* fibers had different lengths and a 3 wt% amount in the fly ash binder content. The investigated amounts of fiber and the ratio between the fiber and binder were selected based on previous research [12,18] and confirmed by preliminary tests. Indeed, lower amounts of fibers impart lower mechanical properties, while higher amounts significantly reduce the workability of the mortars, leading to the formation of macrovoids that negatively affect the mechanical properties. The fibers had been previously subjected to an alkali treatment, and the effect of the treatment was evaluated. A general investigation of the fresh (workability) and cured properties (mechanical and physical) of the mortars was performed. Moreover, with the durability being the most important property to be investigated, water permeability tests of the fiber-modified composites were also performed. The mortars were subjected to freeze–thaw cycles and cyclically stressed by changing both the temperature and the hygrometric conditions.

## 2. Materials and Methods

### 2.1. Materials

#### 2.1.1. Binder

The binding powder was composed of fly ash derived from the plant of Torrevaldaliga (Rome, Italy). It had a density of 2310 kg/m^3^ and a Blaine fineness of 282 m^2^/kg, and the chemical composition determined by inductively coupled plasma (ICP, ICP-OES Serie Optima 3200 XL, PerkinElmer (Waltham, MA, USA)) is reported in Table 1.

#### 2.1.2. Fiber

The giant reed is a *perennial rhizomatous* grass. This plant can be found in all of the temperate areas of Europe. When compared to bamboo, it shows lower but still interesting mechanical properties. The stems were cut to obtain slivers of 30 × 5 × 2 mm. The fibers were recovered by a mechanical procedure. Afterwards, they were soaked in a 4% solution of NaOH for 4 h, washed with distilled water, and cut to the desired size, i.e., 5, 10, and 15 mm in length. The treatment conditions were selected as described in the previously published literature [22,24]. In order to investigate the chemical composition of the fibers and the effect of the chemical treatment, IR and X-ray analyses were performed before and after the treatment.

The crystallinity index (I_c_) of the fibers was determined by X-ray diffraction (XRD) at room temperature, using a Bragg/Brentano diffractometer (Philips PW1710) operating at 40 mA, 40 kV, and λ (Cu-Kα) = 0.154 nm at 0.1°/min in the range of 5–60° Bragg angles. The crystallinity index was calculated by the Buschle-Diller–Zeronian equation:

(1) $$I_{c}=\left(1-\frac{I_{\min}}{I_{\max}}\right)\cdot 100$$

where I_min_ is the intensity at the minimum of the crystalline peak (18° < 2θ < 19°), and I_max_ is the intensity of (002) maximum (20° < 2θ < 25°). The crystallite size and, hence, the structural order of the 002 reflection (L_002_) were calculated according to Scherrer’s equation:

(2) $$L_{002}=\frac{\left(0.91\cdot \lambda \right)}{\left(\beta \cdot \cos\theta \right)}$$

where θ, β, and λ are Bragg’s angle (in degrees), the full width at half-maximum of the 002 reflection, and the wavelength of the X-ray source used, respectively [34]. ATR FT-IR was performed over the wavenumber range 650–4000 cm^−1^ using a PerkinElmer Spectrum One FT-IR spectrometer equipped with a Universal ATR sampling accessory. Sixteen scans were taken for each spectrum, at a resolution of 2 cm^−1^.

#### 2.1.3. Activators

An 8 M molar solution of NaOH (Merck, Darmstadt, Germany) and a Na_2_SiO_3_ (SS, Ingessil, Verona, Italy) solution (SiO_2_/Na_2_O ratio = 2.07, ρ = 1.53 g/cm^3^) were used as activators.

#### 2.1.4. Aggregate

The aggregate used was natural silica sand (SiO_2_ > 96 wt%) with a fixed grain size distribution (d_max_ = 2 mm) conforming to the characteristics of the EN 196-Standard.

### 2.2. Mixing of the Mortars

The alkaline activators were previously mixed and allowed to cool down to room temperature. Afterwards, they were mixed with the fly ash in a Hobart mixer and, once a homogeneous slurry was obtained, natural sand was added. Eventually, the *Arundo* fibers were incorporated into the mortar. The mix design of the composites is reported in Table 2, along with the sample codes that are used hereafter in the text. The ratio between fly ash, activators, and water was set according to previous research [35] to obtain Na_2_O/SiO_2_, SiO_2_/Al_2_O_3_, and Na_2_O/Al_2_O_3_ ratios of 0.12, 3.45, and 0.42, respectively. The fiber volume in the whole composite was about 1.7%. From the fresh mortars, different samples were cast: (a) 20 × 20 × 70 mm prisms to be subjected to physical and mechanical tests, (b) cylinders with 35 mm of diameter and 100 mm height to be subjected to water permeability and durability tests, and (3) 25 × 25 × 280 mm prisms subjected to dimensional stability tests.

### 2.3. Workability

Soon after the mixing process, the mortars were cast in a truncated conical mold with a circular base (100 mm diameter at the bottom, 70 mm diameter at the top, and 60 mm height), according to EN 1015-3. The diameter of the collapsed mortar was recorded after mold removal and shaking.

### 2.4. Physical Tests

The mortar density was determined according to the EN 772-13 Standard after 28 days of curing. The open porosity of the mortars was evaluated by determining the amount of cold water absorbed at atmospheric pressure according to the EN 772-21 Standard after 28 days of curing.

### 2.5. Mechanical Tests

Three-point flexural strength tests, as well as compression tests, were performed at a speed rate of 0.5 mm/min using a Wolpert 100 kN test machine (Wolpert, Neu Ulm, Germany), at 20 ± 1 °C and 65 ± 10 RH%. At least five samples were tested in flexural mode and eight in compressive mode after 28 days of curing at 23 ± 2 °C in sealed plastic bags. Apart from the reported parameters, the procedure followed the instructions of the EN 196-1 Standard, which is formulated for Portland cement composites.

### 2.6. Water Permeability

The water permeability of the composites was evaluated according to the EN 15801 Standard on three cylindrical specimens with 35 mm diameter and 100 mm length, cured for 28 days at 23 ± 1 °C. A Sartorius balance Model CP225D (Sartorius, Gottingen, Germany) was used to evaluate the moisture absorption as a function of time.

### 2.7. Dimensional Stability

The dimensional stability testing was performed according to the ASTM C1012/C1012M Standard on three prisms (25 × 25 × 280 mm) cured at 25 °C and 45 ± 5 RH%.

### 2.8. Freeze–Thaw and Thermo-Hygrometric Cycles

The cylindrical specimens (35 mm diameter and 100 mm height) were subjected to freeze–thaw cycles according to the procedures of the ASTM C666 Standards. The other tests followed the subsequent thermo-hygrometric cycle: (1) 12 h at 10 °C and 95% R.H.; (2) 12 h at 45 °C and 30% R.H., performed by means of a climatic chamber (DY430, Angelantoni, Perugia, Italy). In both experiments, at scheduled times, the dynamic elastic modulus was evaluated as a diagnostic tool for degradation.

### 2.9. Microstructure

The microstructural analysis was performed by means of scanning electron microscopy (SEM XL20 type, FEI Instruments (FEI, Hillsboro, OR, USA) on unperturbed fractured samples obtained after the flexural tests and metalized under vacuum by means of a Quorum 150R ES (Quorum Technologies Ltd., Lewes, UK). The operating conditions were set at 20 kV, and the vacuum condition was below 10^−4^ Torr.

## 3. Results

### 3.1. Fibers’ Characterization

**Figure 1:**
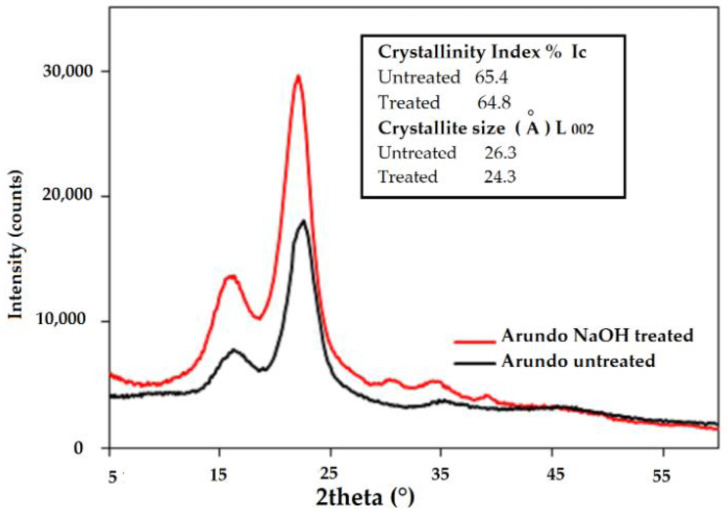
XRD patterns of fiber samples.

The XRD profiles of the treated and untreated fibers exhibited reflections at around 16°, 22°, and 35°, corresponding to the (110), (002), and (004) lattice planes, respectively (Figure 1). Those peaks were assigned to cellulose I, indicating that the chemical treatment did not affect the crystalline form of the cellulose [34]. Additional small peaks were visible around 31 and 39° in the alkali treated fibers. The peak intensity was not affected either, since the crystallinity index—as reported in the figure—remained quite unchanged after the alkali treatment (around 65%), while a small refining of the crystallite size was found (24.3 Å, as opposed to 26.3 Å) after the alkali treatment. In general, alkali treatment is reported to increase the degree of crystallinity because of the removal of non-crystalline materials such as hemicellulose and lignin [36]. On the other hand, it is also reported that this is true only to a certain extent: long exposure to high concentrations of NaOH leads to the weakening of the fiber [37]. Moreover, other authors added that in the case of high concentrations of NaOH, Na^+^ ions can penetrate and swell the crystallites, causing the dissolution of less-ordered crystallites and decreasing the crystallinity index [38].

**Figure 2:**
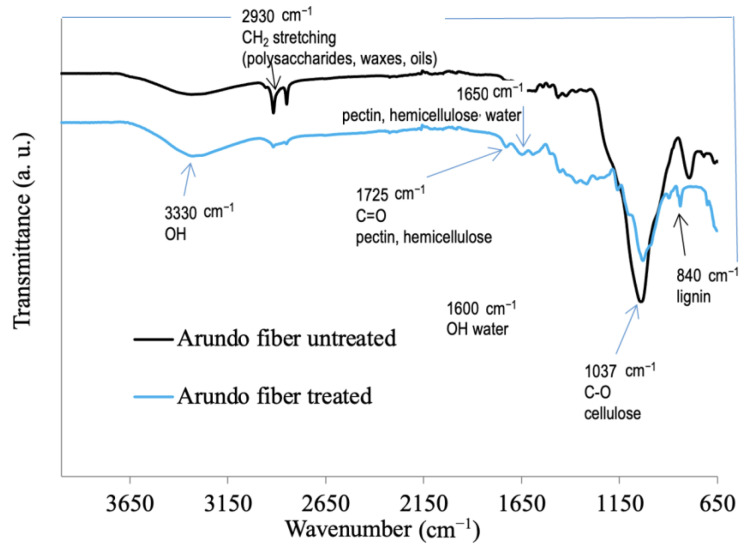
FT-IR profiles of fiber samples.

The FT-IR profiles of the treated and untreated fibers (Figure 2) showed the OH and C-O stretching bands around 3330 and 1037 cm^−1^, respectively, ascribable to polysaccharides and cellulose. CH_2_ stretching was visible around 2930 cm^−1^ and was more intense in the untreated sample, since waxes and oils were removed by the alkali treatment. The treated sample fiber showed a slight increase in the OH band, and a band appeared at around 1650 cm^−1^ (C=O stretching), along with a broad band within the region 1480–1170 cm^−1^, possibly due to lignin, which could have been exposed after the alkali treatment. Similar findings were reported by Elenga et al. [38] for *Raffia textilis* fiber. Loganathan et al. [37] reported that the content of lignin was increased by 10% in NaOH-treated fibers from *Cyrtostachys renda*.

### 3.2. Composites’ Characterization

**Figure 3:**
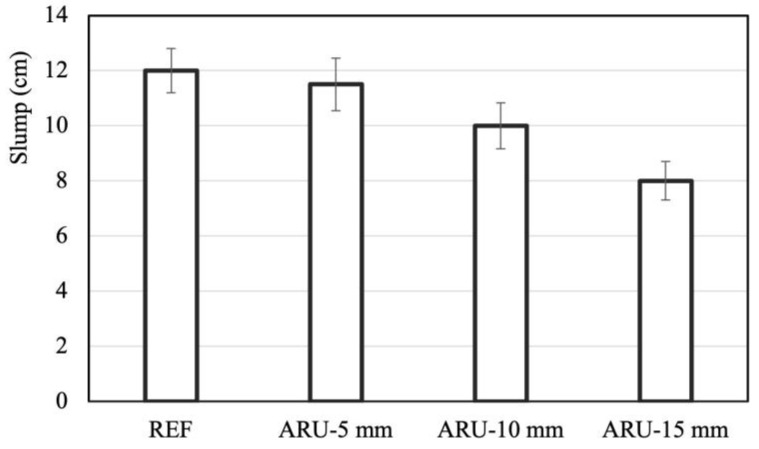
Workability of the investigated mortars.

Figure 3 reports the results of the slump test. The addition of fibers tended to decrease the workability. The reduction was almost negligible in the 5 mm samples, but it reached about 35% in the mortar containing the longest fibers.

The workability data were statistically analyzed with ANOVA, considering statistically distinguishable groups with *p*-values less than 0.05. The analysis showed (Table 3) that the group of specimens REF and the groups of specimens ARU_5 and ARU_10 were not statistically distinguishable, and that the only groups of statistically distinguishable specimens were REF and ARU_15, and ARU_5 and ARU_15 (the table shows the *p*-values for the workability data of the different groups).

In spite of the lower workability, it was nevertheless possible to cast all of the mortars into the molds without creating voids or uneven displacement.

**Figure 4:**
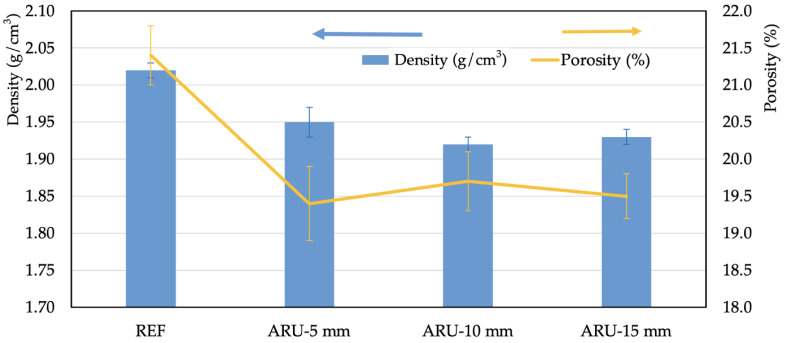
Density and porosity of the investigated mortars.

**Figure 5:**
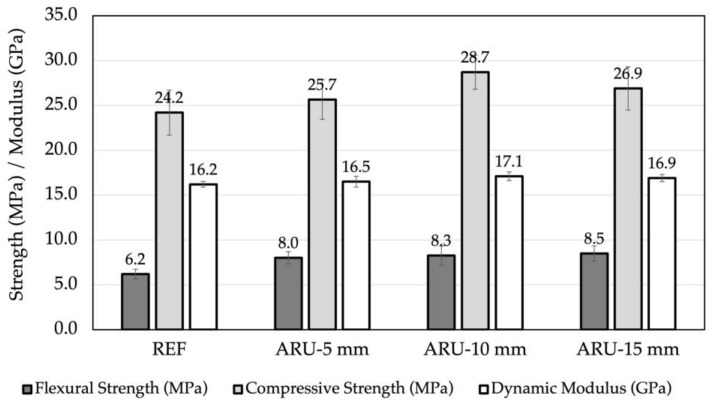
Mechanical properties of the investigated composites after 28 days of curing.

After 28 days of curing, the density and the porosity of the samples were investigated, along with their mechanical properties. The results are summarized in Figure 4 and Figure 5. Slight changes were detected in the values of density and total porosity. The modified mortars had lower density and lower porosity than the REF mortar (Figure 4). This effect was derived from the higher porosity of the alkali-activated matrix when compared to that of the fibers; at the same time, the matrix had a higher density than the fibers and, consequently, a higher value was obtained for the unmodified composite.

Figure 5 reports the results of the mechanical tests (i.e., dynamic elastic modulus, flexural and compressive strength). The value of the dynamic elastic modulus remained almost unchanged in all of the investigated composites, while a large increase in flexural strength (over 30%) took place in the samples modified with *Arundo* fibers. The length of the fibers played a role in the enhancement, although less marked than before. Indeed, a 10% increase was observed upon increasing the fibers’ length. As to the compressive strength, the effect was similar to that observed for the elastic modulus, since a limited increase took place, reaching a maximum value for the 10 mm samples.

**Figure 6:**
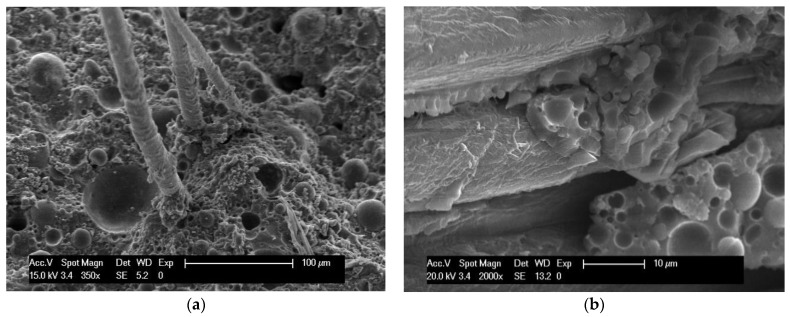
Fracture surface of 10 mm (**a**) and 15 mm (**b**) specimens.

Figure 6 shows the fracture surface of the sample subjected to the flexural test. A fair adhesion between the matrix and the fibers was observed, since no detectable porosities were present at the interphase, and fragments of the matrix were still observable adhering to the *Arundo donax* surface. A possible reaction between the hydroxyl functional groups on the fiber surface and the ones belonging to the developing geopolymeric network has been proposed, and such a reaction may also have taken place in this research, accounting for this effect.

**Figure 7:**
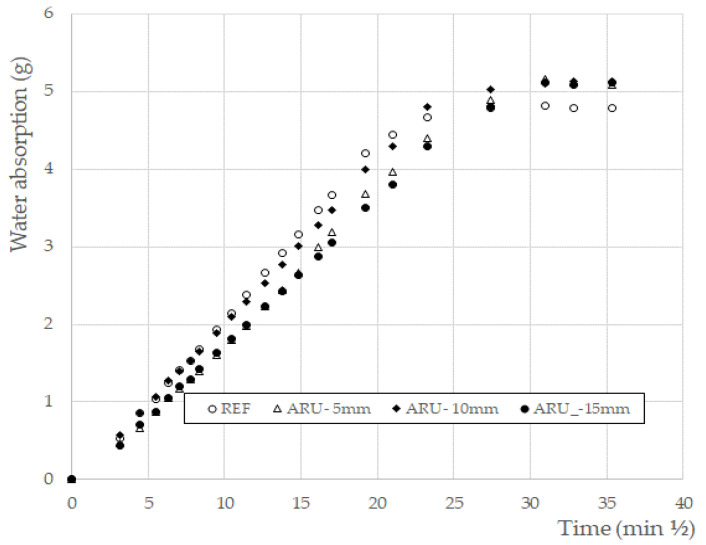
Water permeability.

The results of the permeability test are summarized in Figure 7. The behavior of all of the materials showed a Fickian trend [39], where at the beginning a linear plot was obtained when the water absorption was plotted versus the square root of time. After this, a steady value was reached again for all of the samples. The values remained unchanged at the longest investigated time (4 days; not reported in the figure for sake of clearness). These results are quite important, since they underline that the introduction of the fibers—regardless of their length—does not create preferential paths to the diffusion of water molecules and, consequently, of possible detrimental anions or cations. Moreover, on account of the lower porosity of the fiber compared to that of the matrix, a slight reduction in the initial absorption rate was recorded.

**Figure 8:**
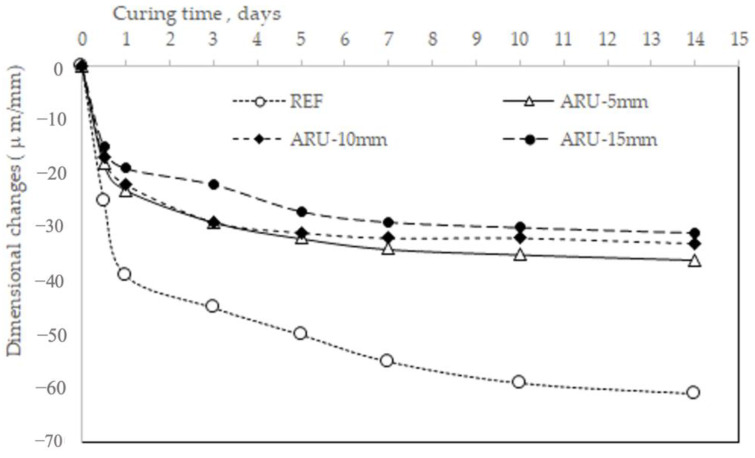
Dimensional stability of the investigated mortars.

Figure 8 shows the dimensional changes in the investigated mortars after demolding. The fibers reduced the shrinkage taking place in the unmodified mortar, and in this case the increase in the *Arundo* fibers’ length seemed to have a positive—albeit limited—effect on increasing the volume stability.

**Figure 9:**
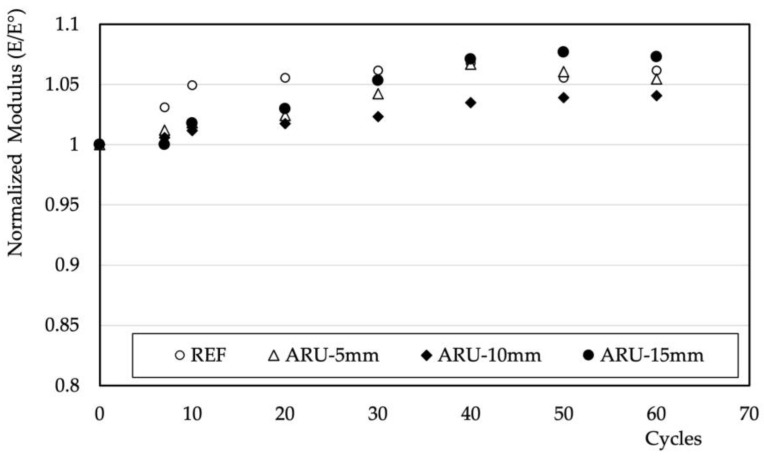
Normalized values of the elastic modulus vs. thermo-hygrometric cycles.

**Figure 10:**
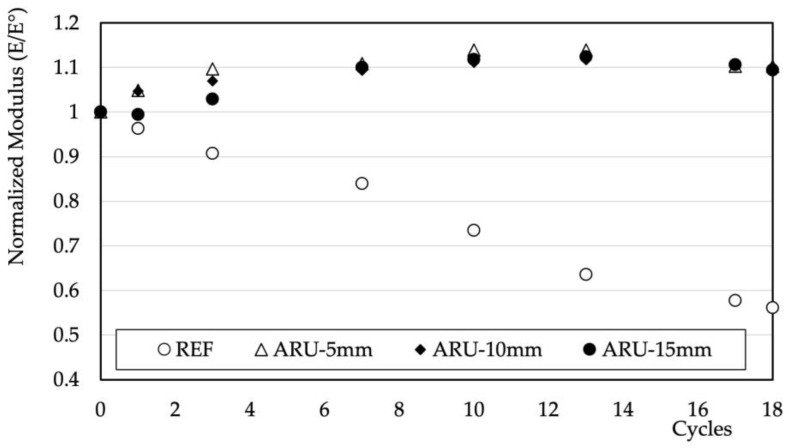
Normalized values of the elastic modulus vs. freeze–thaw cycles.

Both features—that is, the slightly reduced water permeability and the higher dimensional stability hindering the formation of large crevices in the structure—should provide higher durability to the mortars. The results of the environmental stresses deriving from the contemporary changes in temperature and humidity are summarized in Figure 9. The normalized value of the dynamic modulus tended to slightly increase as the number of cycles increased—at least up to the investigated conditions—in all of the investigated samples. These results imply a negligible effect of the mismatch between the organic and hydrophilic nature of the fiber and the inorganic nature of the matrix. The positive effect on the mechanical properties is possibly linked to the enhanced reactivity of the fly ash at higher temperatures (45 °C). Positive results were obtained in other similar materials subjected to wetting and drying cycles [26]. Figure 10 shows the results of the freeze–thaw cycle tests. In these conditions, the behavior of the samples containing *Arundo Donax* differed from that of the unmodified ones. A steady and rapid decrease in the modulus took place in the REF sample. The same rapid decrease in the mechanical properties of unreinforced geopolymers based on fly ash has been evidenced elsewhere [40,41]. All of the samples containing fibers, regardless of their length, showed higher resistance. Indeed, the value of the modulus remained constant at least up to the investigated number of cycles. These natural fibers created the same conditions that have been observed in geopolymeric mortars reinforced with synthetic PVA fibers [42] and POM [43], producing an interlocking effect that offsets the internal stresses caused by water freezing.

## 4. Discussion

The presence of the *Arundo* fibers had positive effects with respect to the mechanical properties of the mortars and their durability. Moreover, they increased the dimensional stability of the mortars after the materials had set. This effect may possibly be even higher than the presently recorded one in the initial period after the mixing process (i.e., before 24 h). Future research should analyze this feature. The initial treatment in alkaline solution did not compromise the crystallinity of the cellulose fraction in the fibers, but it did reduce the presence of undesired substances on their surface (i.e., pectine and waxes), providing a fair adhesion to the matrix. This was evidenced by the increased flexural strength, but also by the morphology of the fracture surfaces and the reduced overall porosity of the modified mortars. The absence of porosities at the interface between the matrix and the fiber was also confirmed by the water permeability test. The results also prove that the fibers do not form a preferential path to the diffusion of the water molecules inside the mortars. Indeed, the initial water uptake rate was slightly lower in the samples containing the fibers, on account of the lower porosity of the fibers themselves. The preliminary tests on the durability of the modified mortars showed that thermal and hygrometric stresses did not compromise the adhesion between the fibers and the matrix, nor did they provoke swelling phenomena. The resistance to freeze–thaw cycles was higher in the modified mortars—a feature that can derive from the higher flexural properties of the mortars.

## 5. Conclusions

In the present study, the effect of the addition of a constant amount of natural fibers with different lengths was investigated. The addition improved almost all of the properties and, at least in the selected range, the fibers’ length proved to be of little consequence for most of the investigated physical, mechanical, and durability properties. In contrast, a significant decrease in workability took place at the highest values. Accordingly, the best trade-off for the material performance was obtained for the medium-length fibers.

## Figures and Tables

**Table 1:** Chemical composition of the fly ash.

	C	O	Na	Mg	Al	Si	K	Ca	Ti	Fe	LOI
wt%	9.4	50.5	0.81	1.08	9.56	21.8	0.99	2.47	0.65	2.68	10.2

**Table 2:** Mix design of the investigated mortars (g).

Sample	Fly Ash	NaOH	Na_2_SiO_3_	Sand	Water	Fiber
REF	100	7.6	38	100	5	3
ARU-5 mm	100	7.6	38	100	5	3
ARU-10 mm	100	7.6	38	100	5	3
ARU-15 mm	100	7.6	38	100	5	3

**Table 3:** ANOVA of the workability results.

*p*-Value	ARU_5	ARU_10	ARU_15
REF	0.879	0.197	0.026
ARU_5		0.399	0.044
ARU_10			0.209

## Data Availability

The authors declare the availability of the data reported in this paper.

## References

[1] Zhang K., Wang K., Liu Z., Ye Z., Zhang B., Lu D., Li L., Xiong Z. (2022). Effect of Magnesium Salt (MgCl_2_ and MgSO_4_) on the Microstructures and Properties of Ground Granulated Blast Furnace Slag (GGBFS)-Based Geopolymer. Materials.

[2] Luhar S., Cheng T.-W., Nicolaides D., Luhar I., Panias D., Sakkas K. (2019). Valorisation of glass wastes for the development of geopolymer composites—Durability, thermal and microstructural properties: A review. Constr. Build. Mater..

[3] Lu N., Ran X., Pan Z., Korayem A.H. (2022). Use of Municipal Solid Waste Incineration Fly Ash in Geopolymer Masonry Mortar Manufacturing. Materials.

[4] Salazar P.A., Fernández C.L., Luna-Galiano Y., Sánchez R.V., Fernández-Pereira C. (2022). Physical, Mechanical and Radiological Characteristics of a Fly Ash Geopolymer Incorporating Titanium Dioxide Waste as Passive Fire Insulating Material in Steel Structures. Materials.

[5] Qaidi S., Tayeh B.A., Isleem H.F., de Azevedo A.R., Ahme H.U., Emad W. (2022). Sustainable utilization of red mud waste (bauxite residue) and slag for the production of geopolymer composites: A review. Case Stud. Constr. Mater..

[6] Trincal V., Multon S., Benavent V., Lahalle H., Balsamo B., Caron A., Bucher R., Caselles L.D., Cyr M. (2022). Shrinkage mitigation of metakaolin-based geopolymer activated by sodium silicate solution. Cem. Concr. Res..

[7] Ohno M., Li V.C. (2014). A feasibility study of strain hardening fiber reinforced fly ash-based geopolymer composites. Constr. Build. Mater..

[8] Matalkah F., Salem T., Shaafaey M., Soroushian P. (2019). Drying shrinkage of alkali activated binders cured at room temperature. Constr. Build. Mater..

[9] Korniejenko K., Łach M., Mikuła J. (2021). The Influence of Short Coir, Glass and Carbon Fibers on the Properties of Composites with Geopolymer Matrix. Materials.

[10] Rashedi A., Marzouki R., Raza A., Rawi N.F.M., Naveen J. (2021). Mechanical, Fracture, and Microstructural Assessment of Carbon-Fiber-Reinforced Geopolymer Composites Containing Na_2_O. Polymers.

[11] Le C.H., Louda P., Buczkowska K.E., Dufkova I. (2021). Investigation on Flexural Behavior of Geopolymer-Based Carbon Textile/Basalt Fiber Hybrid Composite. Polymers.

[12] Manzi S., Lancellotti I., Masi G., Saccani A. (2020). Alkali-Activated Binders From Waste Incinerator Bottom Ashes and Metakaolin Reinforced by Recycled Carbon Fiber Composites. Front. Mater..

[13] Deng Z., Yang Z., Bian J., Lin J., Long Z., Hong G., Yang Z., Ye Y. (2022). Advantages and disadvantages of PVA-fibre-reinforced slag- and fly ash-blended geopolymer composites: Engineering properties and microstructure. Constr. Build. Mater..

[14] Assaedi H., Alomayri T., Siddika A., Shaikh F., Alamri H., Subaer S., Low I.-M. (2019). Effect of nanosilica on mechanical prop-erties and microstructure of PVA fiber-reinforced geopolymer composite (PVA-FRGC). Materials.

[15] Silva G., Kim S., Aguilar R., Nakamatsu J. (2020). Natural fibers as reinforcement additives for geopolymers—A review of potential eco-friendly applications to the construction industry. Sustain. Mater. Technol..

[16] Liu D., Song J., Anderson D.P., Chang P.R., Hua Y. (2012). Bamboo fiber and its reinforced composites: Structure and properties. Cellulose.

[17] Maier M., Javadian A., Saeidi N., Unluer C., Taylor H.K., Ostertag C.P. (2020). Mechanical Properties and Flexural Behavior of Sustainable Bamboo Fiber-Reinforced Mortar. Appl. Sci..

[18] Saccani A., Molari L., Totaro G., Manzi S. (2021). Geopolymers Reinforced with Natural Fibers: A Comparison among Different Sources. Appl. Sci..

[19] Lv C., Liu J., Guo G., Zhang Y. (2022). The Mechanical Properties of Plant Fiber-Reinforced Geopolymers: A Review. Polymers.

[20] Camargo M.M., Taye E.A., Roether J.A., Redda D.T., Boccaccini A.R. (2020). A Review on Natural Fiber-Reinforced Geopolymer and Cement-Based Composites. Materials.

[21] de Azevedo A.R.G., Cruz A.S.A., Marvila M.T., de Oliveira L.B., Monteiro S.N., Vieira C.M.F., Fediuk R., Timokhin R., Vatin N., Daironas M. (2021). Natural Fibers as an Alternative to Synthetic Fibers in Reinforcement of Geopolymer Matrices: A Comparative Review. Polymers.

[22] Chikouche M.D.L., Merrouche A., Azizi A., Rokbi M., Walter S. (2015). Influence of alkali treatment on the mechanical properties of new cane fibre/polyester composites. J. Reinf. Plast. Compos..

[23] Mwaikambo L.Y., Ansell M.P. (2022). Chemical modification of hemp, sisal, jute, and kapok fibers by alkalization. J. Appl. Polym. Sci..

[24] Wang F., Zhou S., Li L., Zhang X. (2018). Changes in the morphological-mechanical properties and thermal stability of bamboo fibers during the processing of alkaline treatment. Polym. Compos..

[25] Assaedi H., Alomayri T., Shaikh F., Low I.-M. (2019). Influence of Nano Silica Particles on Durability of Flax Fabric Reinforced Geopolymer Composites. Materials.

[26] dos Santos G.Z.B., de Oliveira D.P., Filho J.D.A.M., da Silva N.M. (2021). Sustainable geopolymer composite reinforced with sisal fiber: Durability to wetting and drying cycles. J. Build. Eng..

[27] Gholampour A., Danish A., Ozbakkaloglu T., Yeon J.H., Gencel O. (2022). Mechanical and durability properties of natural fiber-reinforced geopolymers containing lead smelter slag and waste glass sand. Constr. Build. Mater..

[28] Moujoud Z., Sair S., Ousaleh H.A., Ayouch I., El Bouari A., Tanane O. (2023). Geopolymer composites reinforced with natural Fibers: A review of recent advances in processing and properties. Constr. Build. Mater..

[29] Molari L., Coppolino F.S., García J.J. (2021). *Arundo donax*: A widespread plant with great potential as sustainable structural material. Constr. Build. Mater..

[30] Mentrasti L., Molari L., Fabiani M. (2021). Poisson’s ratio bounds in orthotropic materials. Application to natural composites: Wood, bamboo and Arundo donax. Compos. Part B Eng..

[31] Fiore V., Scalici T., Valenza A. (2014). Characterization of a new natural fiber from *Arundo donax* L. as potential reinforcement of polymer composites. Carbohydr. Polym..

[32] Bessa W., Trache D., Derradji M., Ambar H., Benziane M., Guedouar B. (2021). Effect of different chemical treatments and loadings of *Arundo donax* L. fibers on the dynamic mechanical, thermal, and morphological properties of bisphenol A aniline based polybenzoxazine composites. Polym. Compos..

[33] Ferrandez-García A.A., Ortuño T.G., Ferrandez-Villena M., Ferrandez-Garcia A., Ferrandez-García M.T. (2022). Evaluation of Particleboards Made from Giant Reed (*Arundo donax* L.) Bonded with Cement and Potato Starch. Polymers.

[34] Sisti L., Totaro G., Vannini M., Fabbri P., Kalia S., Zatta A., Celli A. (2016). Evaluation of the retting process as a pre-treatent of vegetable fibres for the preparation of high-performance polymer biocomposites. Ind. Crops Prod..

[35] Carabba L., Santandrea M., Carloni C., Manzi S., Bignozzi M.C. (2017). Steel fiber reinforced geopolymer matrix (S-FRGM) composites applied to reinforced concrete structures for strengthening applications: A preliminary study. Compos. Part B Eng..

[36] Bessa W., Trache D., Derradji M., Ambar H., Tarchoun A.F., Benziane M., Guedouar B. (2020). Characterization of raw and treated *Arundo donax* L. cellulosic fibers and their effect on the curing kinetics of bisphenol A-based benzoxazine. Int. J. Biol. Macromol..

[37] Loganathan T.M., Sultan M.T.H., Ahsan Q., Jawaid M., Naveen J., Shah A.U.M., Hua L.S. (2020). Characterization of alkali treated new cellulosic fibre from *Cyrtostachys renda*. J. Mater. Res. Technol..

[38] Elenga R.G., Djemia P., Tingaud D., Chauveau T., Maniongui J.G., Dirras G. (2013). Effects of Alkali Treatment on the Microstructure, Composition, and Properties of the Raffia textilis Fiber. Bioresources.

[39] Babaee M., Castel A. (2018). Water vapor sorption isotherms, pore structure, and moisture transport characteristics of alkali-activated and Portland cement-based binders. Cem. Concr. Res..

[40] Zhao R., Yuan Y., Cheng Z., Wen T., Li J., Li F., Ma Z.J. (2019). Freeze-thaw resistance of Class F fly ash-based geopolymer concrete. Constr. Build. Mater..

[41] Jiao Z., Li X., Yu Q. (2021). Effect of curing conditions on freeze-thaw resistance of geopolymer mortars containing various calcium resources. Constr. Build. Mater..

[42] Li F., Chen D., Lu Y., Zhang H., Li S. (2022). Influence of mixed fibers on fly ash based geopolymer resistance against freeze-thaw cycles. J. Non-Cryst. Solids.

[43] Shi X., Wang X., Wang Q., Zhang T., Yang F., Xu Y., Zhan J. (2023). Experimental Analysis and Establishment of Strength Attenuation Model of POM Fiber Reinforced Geopolymeric Recycled Concrete under Freeze-Thaw Cycles. Materials.

